# Influence of Diabetes Mellitus on Metabolic and Hormonal Interactions Promoting Aggressive Prostate Cancer

**DOI:** 10.7759/cureus.96338

**Published:** 2025-11-07

**Authors:** Afreen Khan, Anu Chandra, Preeti Agarwal, Syed Tasleem Raza, Avneet Gupta, Abbas A Mahdi, Satya N Sankhwar

**Affiliations:** 1 Department of Biochemistry, Era's Lucknow Medical College and Hospital, Era University, Lucknow, IND; 2 Department of Pathology, King George's Medical University, Lucknow, IND; 3 Department of Urology, King George's Medical University, Lucknow, IND; 4 Department of Biochemistry, King George's Medical University, Lucknow, IND

**Keywords:** benign prostatic hyperplasia, diabetes mellitus, dyslipidemia, gleason score, insulin, insulin-like growth factor, prostate cancer, prostate specific antigen

## Abstract

Background and objectives

Diabetes mellitus might play a vital role in promoting prostate cancer development, progression, and aggressiveness. The influence of diabetes on prostate cancer outcomes has long been debated. However, the mechanism through which diabetes mediates its effect on prostate cancer development and progression has not been established. This study aimed to study the influence of diabetes mellitus on prostate cancer outcome and aggressiveness.

Material and methods

This study includes 100 patients newly diagnosed with benign prostatic hyperplasia (BPH) and 200 patients with histologically confirmed prostate adenocarcinoma, of which 100 were diabetic. Descriptive statistics were performed to analyse and summarize the results as mean±standard deviation (SD). Statistically significant differences between two or more groups were determined using ANOVA and the post hoc Tukey test. Chi-square test was performed to compare the distribution of tumor grades and risk categories between patients with prostate cancer with diabetes and without diabetes. Correlation analyses were performed to analyse the association of metabolic and hormonal parameters among themselves and cancer grade. Multiple linear regression analysis was done to analyse the relationship of hormone profile with the risk of prostate cancer in men with and without diabetes.

Results

Data showed that serum insulin and IGF-1 were significantly elevated in prostate cancer, highest being in prostate cancer with diabetes, and they had a positive association with prostate cancer Gleason score, grade, and high risk. Lipid profile and HbA1c also had a significant association with prostate cancer Gleason score and grade. Men with diabetes and prostate cancer were diagnosed with higher-grade and high-risk cancer than men with prostate cancer but without diabetes.

Conclusion

This study confirms the influence of diabetes on metabolic and hormonal parameters, particularly serum HbA1c, insulin, IGF1, and prostate-specific antigen, which are positively associated with higher grade and high risk, thus leading to the development and promotion of aggressive cancer in patients with coincidental diabetes.

## Introduction

According to the Global Cancer Observatory (GLOBOCAN 2022), prostate cancer is the most frequently diagnosed cancer in 118 countries and the second frequently diagnosed cancer in 33 countries, and the fifth leading cause of death worldwide [1]. In the last decade, several studies highlighted the role of diabetes in prostate cancer development and progression; however, the association is yet to be explored as existing reports reflect contradictory findings. Some epidemiological studies reported decreased risk [2,3], whereas some reported increased risk [4,5] of prostate cancer with coincidental diabetes.

Prostate-specific antigen (PSA) is a diagnostic biomarker for prostate cancer, whose regulation is mediated by the binding of testosterone to androgen receptor (AR) [6]. In recent years, studies confirmed that male patients with diabetes are more prone to develop hypogonadism, thus low testosterone levels, leading to low *PSA* gene and protein expression [7]. Men with diabetes also exhibit insulin resistance, which leads to an elevation in the serum levels of insulin and insulin-like growth factor-1 (IGF-1), promoting cell proliferation and survival in various cancers, including prostate cancer [8]. Most previous studies have reported more aggressive prostate cancer with increased lymph node metastasis in men with diabetes [9]. However, one recent study reported the incidence of smaller, less aggressive prostate cancer in men with diabetes [10].

Based on all these premises and considering that diabetes is characterized by hyperglycemia, hyperlipidemia, hyperinsulinemia, and insulin resistance, we hypothesize that diabetes modulates serum PSA, insulin, IGF-1, testosterone, and lipid profile in men with prostate cancer, leading to both late detection and cancer progression. Therefore, current research aims to identify the influence of diabetes on lipid profile, serum PSA, and endocrine factors such as insulin, IGF-1, and testosterone in prostate cancer and its association with progression, risk, and aggressiveness of prostate cancer.

This study was published as a Preprint (DOI:10.2139/ssrn.5385346) on the SSRN preprint repository on August 13, 2025.

## Materials and methods

Study design and population

This comparative case series recruited 300 individuals from June 2020 to March 2024, divided into three study groups: 100 patients with prostate cancer along with diabetes (PCa+DM), 100 patients with prostate cancer but without diabetes (PCa-DM), and 100 BPH patients (comparison group) who underwent transurethral resection of prostate (TURP) at the Department of Urology, King George’s Medical University (KGMU), Lucknow, Uttar Pradesh, India. Newly diagnosed, treatment-naïve patients were included. Those with a history of prostatitis, coronary artery disease, hepatic or renal diseases, or those receiving testosterone replacement therapy or androgen deprivation therapy were excluded.

Sample size was calculated on the basis of the odds ratio of risk of high serum IGF-1 (OR=3.35) on prostate cancer using the formula: $n = \frac{(Z_{\alpha} + Z_{\beta})^2}{[\ln(1 - e)]^2} \left[ \frac{1 - p_1}{p_1} + \frac{1 - p_2}{p_2} \right]$, where Z_α_: standard normal deviate for significance level α (0.05), Z_β_: standard normal deviate for power (1−β), e: the risk ratio under the research hypothesis (e=0.5), and p1,p2: proportions in two groups, assuming power = 0.90, which yielded a minimum of 114 participants across three groups. We recruited 300 participants to account for potential data loss.

In this study, age was identified as a confounding factor due to the reported incidence of prostate cancer at a significantly higher age than BPH. As direct matching was not feasible, age was adjusted for its potential confounding effects in the statistical tests. Diabetes mellitus was diagnosed by a physician based on American Diabetes Association criteria: fasting blood glucose (FBG) ≥126 mg/dL, HbA1c ≥6.5%.

Diagnosis of BPH and prostate cancer

To rule out prostate cancer, a 12-core transrectal ultrasound-guided (TRUS) biopsy (also called a needle biopsy) was performed by expert urologists before TURP on suspected patients with PSA levels >4ng/ml. The samples were sent to the Department of Pathology, KGMU, for histopathological examination by an expert pathologist for confirmatory diagnosis of prostate cancer.

Grading and staging of prostate cancer were done using the gold standard Gleason score (GS) system by assessing the architectural pattern of cancer cells and translating into the International Society of Urological Pathology (ISUP) grading system [11], ranging from 1 to 5. Risk group was assigned according to the National Comprehensive Cancer Network (NCCN) guideline [12], European Association of Urology (EAU) guidelines [13], and American Urological Association (AUA) guideline [14], which classify prostate cancer into low, intermediate, and high-risk groups.

Variables collected

Clinical data, including medical history of diabetes, hypertension, and medications, were obtained from all participants. In addition, family history of prostate cancer and personal history, including diet, smoking, alcohol consumption, and exercise status, were also taken based on a standardized questionnaire and interview. Body mass index (BMI) was calculated using the formula: weight (kg)/height^2^ (m^2^).

Specimen collection and laboratory assays

For all participants, 2 mL of fasting blood samples were collected in plain vials. Serum was separated and stored at −80°C until use. Estimation of serum lipid (total cholesterol, high-density lipoprotein cholesterol (HDL‑C), and triglycerides) was performed enzymatically on a semi-autoanalyzer. Values of very low‑density lipoprotein‑cholesterol (VLDL‑C) and low-density lipoprotein cholesterol (LDL‑C) were calculated using Friedwald’s formula. Enzyme-linked immunosorbent assay (ELISA) kits were used to estimate the concentration of serum Insulin (DRG EIA-2935), IGF-1 (DRG EIA-4140), and testosterone (DRG EIA-1559) according to the manufacturer’s protocol (DRG Diagnostics GmbH, Marburg, Germany). Serum PSA levels, FBG, and HbA1c levels were evaluated from hospital records.

Statistical analysis

Analysis of data and result interpretation were done using descriptive statistics to summarize and organize datasets and compared among study groups. Qualitative variables were represented as numbers and relative frequency (%), whereas quantitative variables were represented as mean ± standard deviation (SD). Statistically significant differences among BPH, PCa-DM, and PCa+DM groups were determined using the ANOVA test, and the pairwise differences were identified using the Tukey post hoc test for parameters that showed significant differences in groupwise analysis to further understand which specific group differs significantly from the others. Multiple linear regression analysis was done to assess the relationship of hormonal profile and serum PSA with the risk of prostate cancer in men with diabetes and men without diabetes. Pearson’s correlation was performed in each study group to analyse the association of metabolic and hormonal parameters among each other. Spearman’s correlation analysis was performed to analyse the association of study parameters with cancer grade and risk in both prostate cancer groups. Multiple linear regression analysis was performed to find the relationship of serum PSA, insulin, IGF-1, and testosterone with prostate cancer in the absence and presence of coincidental diabetes, considering BPH as the reference group. The effect size was calculated based on Cohen’s guidelines to understand the proportion of variance in the dependent variable. Diagnostic plots, including actual vs predicted plot, residual vs predicted plot, residual vs observation order plot, and QQ (quantile-quantile) plot, were examined to assess the assumption of multiple linear regression. The association of diabetes with prostate cancer grade risk and aggressiveness was determined by Chi Chi-Square Test. All statistical analyses were performed using IBM SPSS Statistics for Windows, version 26 (IBM Corp., Armonk, New York, United States) and Graphpad Prism v10 (Dotmatics, Boston, Massachusetts, United States). The significance level was taken as p<0.05.

Ethics statement

The study was conducted in accordance with the Code of Ethics of the World Medical Association, Declaration of Helsinki. The study protocol was approved by the Institutional Ethics Committees of Era's Lucknow Medical College and Hospital and King George's Medical University (approval letters: ELMC&H/R_Cell/EC/2020/37, ELMC&H/R_Cell/EC/2020/121A, and 520/Ethics/2020). Samples were collected after obtaining written informed consent from each study participant.

## Results

Baseline characteristics

The distribution of cases among the three study groups, PCa+DM, PCa-DM, and BPH, is equal (n=100). The comparison of baseline characteristics among the three study groups shows several significant findings (Table 1). The mean age differed significantly among study groups (F=9.47, p<0.001). Significant difference was found between PCa+DM vs BPH (p = 0.020) and PCa-DM vs BPH (p < 0.001). However, the difference was not significant between the two PCa groups (p = 0.242). BMI showed no significant differences (p=0.369).

**Table 1:** Groupwise descriptive summary and comparison of baseline, metabolic, and hormonal parameters Bold values represent significant results (p<0.05). ^a^F-value (ANOVA) ^b^Chi sq. PCa+DM: prostate cancer with diabetes mellitus; PCa-DM: prostate cancer without diabetes mellitus; BPH: benign prostatic hyperplasia; BMI: body mass index; HTN: hypertension, T.CHO: total cholesterol; TG: triglyceride; HDL: high density lipoprotein; LDL: low density lipoprotein; VLDL: very low density lipoprotein; HbA1c: glycated hemoglobin, FBG: fasting blood glucose; PSA: prostate-specific antigen; IGF-1: insulin-like growth factor-1

Study Variables		PCa+DM	PCa-DM	BPH	F-value (ANOVA)^a ^ Chi sq. ^b^	p-value
Age (years), mean±SD		68.21±9.04	70.24±8.40	64.82±9.24	9.47^a^	<0.001
BMI (kg/m^2^), mean±SD		22.40±3.39	21.88±3.14	22.53±3.75	1.00^a^	0.369
Family history, n (%)	Yes	27 (27%)	20 (20%)	15 (15%)	4.43^b^	0.109
	No	73 (73%)	80 (80%)	85 (85%)		
Smoking status, n (%)	Yes	25 (25%)	30 (30%)	33 (33%)	1.58^b^	0.455
	No	75 (75%)	70 (70%)	67 (67%)		
Alcohol intake, n (%)	Yes	16 (16%)	33 (33%)	29 (29%)	8.21^b^	0.016
	No	84 (84%)	67 (67%)	71 (71%)		
Physical activity, n (%)	Not active	26 (26%)	26 (26%)	17 (17%)	3.50^b^	0.478
	Moderately active	61 (61%)	63 (63%)	66 (66%)		
	Very active	13 (13%)	11 (11%)	16 (16%)		
Diet	Non-Veg	12 (12%)	27 (27%)	18 (18%)	7.41^b^	0.025
	Veg	88 (88%)	73 (73%)	82 (8%)		
Hypertension	Yes	16 (16%)	36 (36%)	33(33%)	11.46^b^	0.003
	No	84 (84%)	64 (64%)	67 (67%)		
T. CHO (mg/dl), mean±SD		194.63±43.17	186.35±39.89	183.68±50.79	1.62	0.199
TG (mg/dl), mean±SD		174.35±52.23	169.98±56.83	156.63±54.54	2.86	0.059
HDL (mg/dl), mean±SD		43.90±7.79	46.82±7.99	50.17±6.50	17.71	<0.001
LDL (mg/dl), mean±SD		115.86±41.53	105.53±39.39	102.18±47.97	2.73	0.067
VLDL (mg/dl), mean±SD		34.87±10.45	34.00±11.37	31.33±10.91	2.86	0.059
HbA1c (mmol/L) mean±SD		6.83±0.60	5.52±0.40	5.13±0.29	386.57	<0.001
FBG (mg/dl), mean±SD		130.62±12.14	97.09±7.47	91.84±8.45	483.68	<0.001
PSA (ng/ml) mean±SD		22.38±18.62	39.72±32.25	6.30±5.91	58.95	<0.001
Insulin (µIU/ml) mean±SD		20.67±7.66	16.40±8.32	14.39±4.35	21.02	<0.001
IGF-1 (ng/ml) mean±SD		354.58±29.48	352.22±30.04	331.11±32.28	17.81	<0.001
Testosterone (ng/ml) mean±SD		3.05±1.48	3.15±1.22	3.85±1.78	8.35	<0.001

A positive family history for prostate cancer was not significantly different among study groups (p = 0.109). Nevertheless, a higher frequency was observed in both PCa groups compared to BPH. Smoking status was also similar among groups (p = 0.455). However, a significant difference in alcohol consumption was observed among the groups (p = 0.016), with the highest proportion observed in PCa-DM, followed by BPH and PCa+DM. Physical activity status was also similar among groups (p = 0.478), with most participants being moderately active in all groups. Dietary habits revealed a significant variation (p = 0.025), with non-vegetarian diets being most common in PCa-DM compared to BPH and PCa+DM. The history of hypertension (HTN) prevalence was also significantly different across the groups (p = 0.003), being most prevalent in both PCa groups compared to BPH.

Metabolic and hormonal profiles

The levels of metabolic and hormonal parameters compared among three study groups (Table 1) and analysis of pairwise differences among three groups (PCa+DM vs PCa-DM, PCa+DM vs BPH, and PCa-DM vs BPH) (Table 2) show several significant findings. Serum levels of total cholesterol, triglyceride (TG), LDL, and VLDL were found to be highest in the PCa+DM group compared to the PCa-DM group, and lowest values were observed in the BPH group, but the difference was not significant across study groups (p>0.05). However, serum HDL levels showed a significant difference among all the study groups (p<0.001). In pairwise comparisons, the BPH group had higher HDL levels compared to PCa+DM (p = 0.016) and PCa-DM (p < 0.001). Comparison of HDL levels between both PCa groups showed lower HDL levels in the PCa+DM group than the PCa-DM group (p = 0.005). Similar observations were found for glycemic parameters: FBG and HbA1c, where all three groups show significant differences with each other (p < 0.001), the highest value being observed in the PCa+DM group, which gradually decreased in the PCa-DM group, and the lowest in the BPH group.

**Table 2:** Tukey post hoc paired comparisons of metabolic and hormonal parameters Bold values represent significant results (p<0.05). PCa+DM: prostate cancer with diabetes mellitus; PCa-DM: prostate cancer without diabetes mellitus; BPH: benign prostatic hyperplasia; HDL: high density lipoprotein; HbA1c: glycated hemoglobin, FBG: fasting blood glucose; PSA: prostate-specific antigen; IGF-1: insulin-like growth factor-1

Parameters	PCa+DM vs PCa-DM		PCa+DM vs BPH		PCa-DM vs BPH	
	Mean Diff.	p-value	Mean Diff.	p-value	Mean Diff.	p-value
AGE, years (Mean±SD)	-2.03	0.242	3.39	0.020	5.42	<0.001
HDL, mg/dl (Mean±SD)	-2.92	0.016	-6.27	<0.001	-3.35	0.005
HbA1c, mmol/L (Mean±SD)	1.31	<0.001	1.70	<0.001	0.39	<0.001
FBG, mg/dl (Mean±SD)	33.53	<0.001	38.79	<0.001	5.26	<0.001
PSA, ng/ml (Mean±SD)	-17.34	<0.001	16.08	<0.001	33.43	<0.001
Insulin, µIU/ml (Mean±SD)	4.28	<0.001	6.28	<0.001	2.01	0.108
IGF-1, ng/ml (Mean±SD)	2.36	0.849	23.47	<0.001	21.11	<0.001
Testosterone, ng/ml (Mean±SD)	-0.10	0.896	-0.80	0.001	-0.70	0.003

Serum PSA levels were markedly elevated in PCa-DM compared to PCa+DM and BPH, with the difference being highly significant (F = 58.95, p < 0.001). Insulin levels also showed significant variation (F = 21.02, p < 0.001) with the highest levels in PCa+DM, followed by PCa-DM and BPH. Similarly, IGF-1 levels were significantly higher in the PCa+DM and PCa-DM groups compared to BPH (F = 17.81, p < 0.001). Testosterone levels were significantly lowest in PCa+DM and highest in BPH (F = 8.35, p < 0.001).

Relationship of serum levels of insulin, IGF-1, PSA, and testosterone with prostate cancer

**Figure 1:**
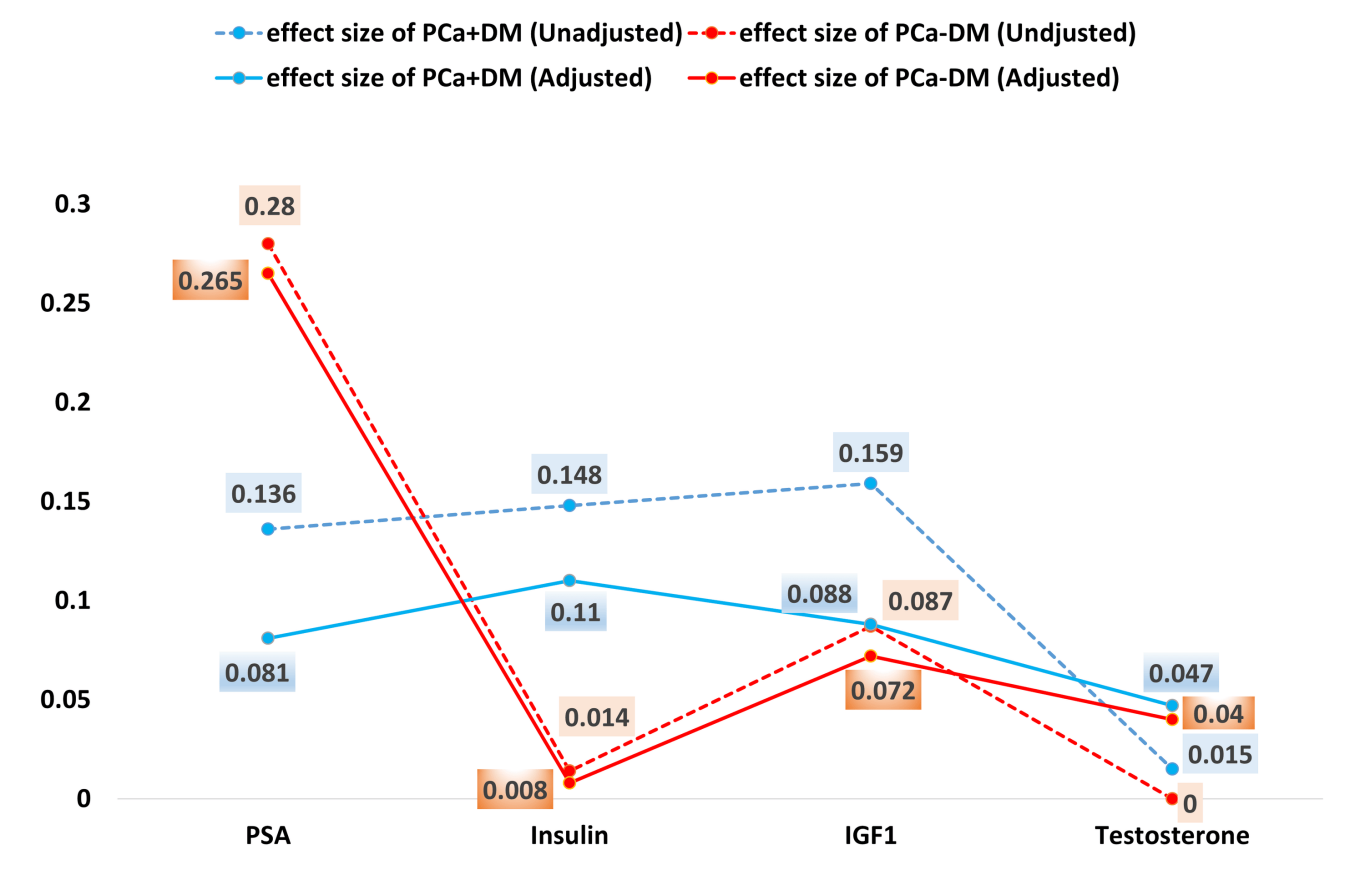
Effect size based on Cohen’s guidelines showing strong effect of diabetes on serum PSA, insulin and IGF-1 levels in prostate cancer patients. Bold values represent significant results (p<0.05). PCa+DM: prostate cancer with diabetes mellitus; PCa-DM: prostate cancer without diabetes mellitus; PSA: prostate-specific antigen; IGF-1: insulin-like growth factor-1

The influence of diabetes condition on various biomarkers demonstrated significant differences across study groups in multiple linear regression analysis (Table 3, Figure 1). For PSA levels, both the PCa+DM (adjusted B = 15.82, p < 0.001, effect size = 0.018) and the PCa-DM (B = 32.65, p < 0.001, effect size = 0.265) showed significantly higher values compared to the BPH (reference group). Insulin levels were also significantly elevated in the PCa+DM (Adjusted B = 6.02, p < 0.001, effect size = 0.110), while the PCa-DM group did not show a statistically significant difference (p = 0.116). IGF-1 levels were significantly higher in both the PCa+DM (Adjusted B = 23.35, p < 0.001, effect size = 0.088) and PCa-DM (Adjusted B = 21.326, p < 0.001, effect size = 0.072) compared to BPH. Testosterone levels were significantly lower in both the PCa+DM (Adjusted B = -0.82, p < 0.001, effect size = 0.047) and PCa-DM (Adjusted B = -0.77, p < 0.001, effect size = 0.040) compared to BPH.

**Table 3:** Linear regression analysis showing relationship of serum levels of insulin, IGF-1, PSA, and testosterone with PCa+DM and PCa-DM groups Bold values represent significant results (p<0.05); ^a^Unadjusted; ^b^Adjusted for age and BMI PCa+DM: Prostate cancer with diabetes mellitus; PCa-DM: prostate cancer without diabetes mellitus; BPH: benign prostatic hyperplasia; B: beta coefficient, SE: standard error PSA: prostate-specific antigen; IGF-1: insulin-like growth factor-1

Dependent Variable	Influencer	B	SE	t-value	p-value	effect size
PSA	Intercept	6.31^a^	4.55	1.39	0.169	0.019
		21.77^b^	12.36	1.76	0.079	0.010
	PCa + DM	25.36^a^	6.43	3.94	<0.001	0.136
		15.82^b^	3.10	5.10	<0.001	0.081
	PCa - DM	39.92^a^	6.43	6.21	<0.001	0.280
		32.65^b^	3.17	10.31	<0.001	0.265
	BPH	Ref.				
Insulin	Intercept	14.97^a^	1.32	11.31	<0.001	0.564
		7.626^b^	3.98	1.92	0.056	0.012
	PCa + DM	7.76^a^	1.87	4.14	<0.001	0.148
		6.02^b^	0.10	6.02	<0.001	0.110
	PCa - DM	2.21^a^	1.87	1.18	0.242	0.014
		1.61^b^	1.02	1.58	0.116	0.008
	BPH	Ref.				
IGF-1	Intercept	344.15^a^	4.13	83.25	<0.001	0.986
		305.50^b^	17.44	17.52	<0.001	0.510
	PCa + DM	25.29^a^	5.85	4.33	<0.001	0.159
		23.35^b^	4.37	5.34	<0.001	0.088
	PCa - DM	17.99^a^	5.85	3.08	0.003	0.087
		21.326^b^	4.47	4.77	<0.001	0.072
	BPH	Ref.				
Testosterone	Intercept	3.27^a^	0.25	12.98	<0.001	0.630
		5.19^b^	0.86	6.07	<0.001	0.111
	PCa + DM	-0.43^a^	0.36	-1.21	0.230	0.015
		-0.82^b^	0.22	-3.82	<0.001	0.047
	PCa - DM	-0.05^a^	0.36	-0.13	0.899	0.000
		-0.77^b^	0.22	-3.50	<0.001	0.040
	BPH	Ref.				

**Figure 2:**
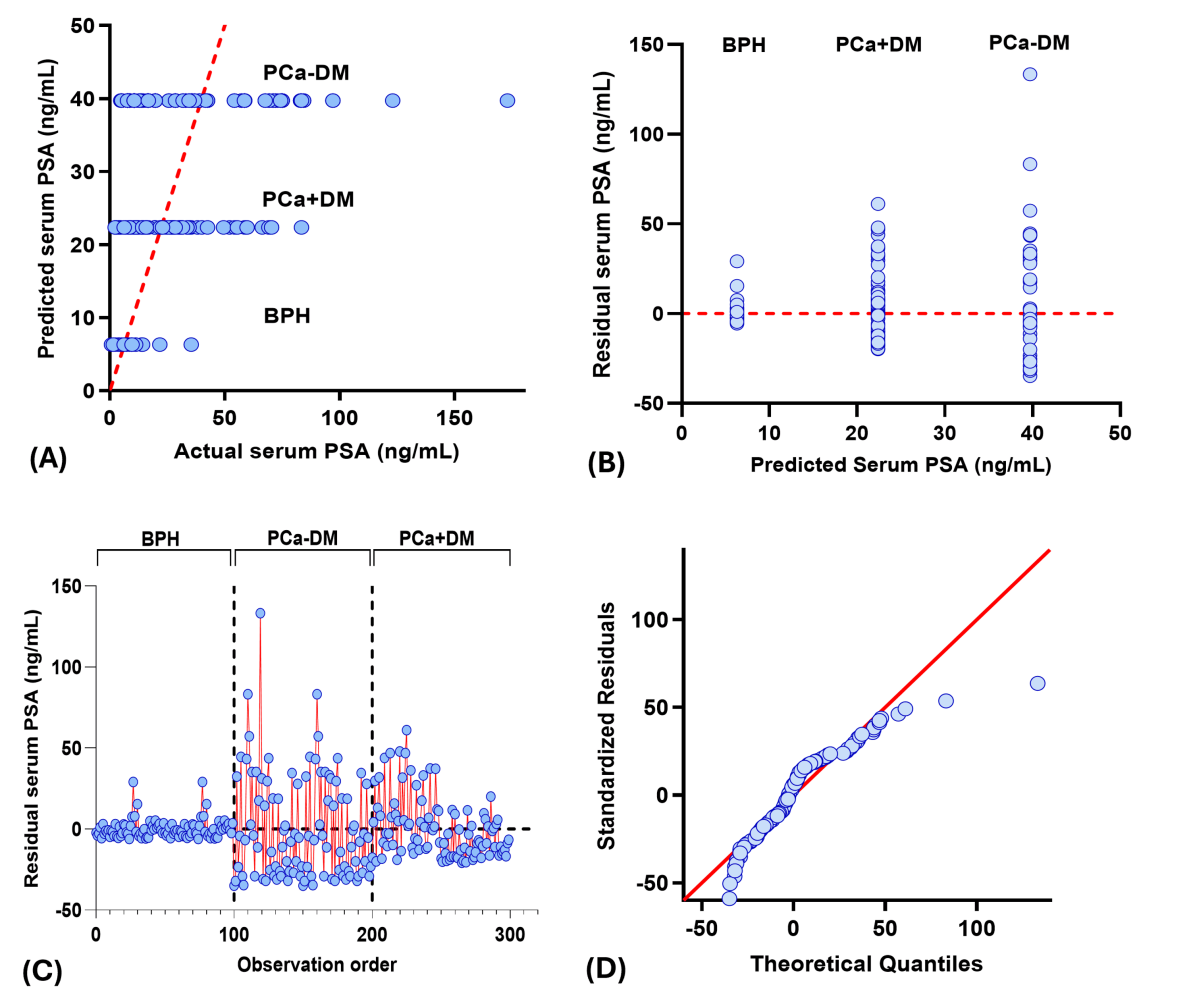
Diagnostic plot for PSA multiple regression analysis (A) Observed vs predicted plot, (B) Residual vs predicted plot, (C) Observation order vs residuals, (D) QQ plot. PCa+DM: prostate cancer with diabetes mellitus; PCa-DM: prostate cancer without diabetes mellitus; BPH: benign prostatic hyperplasia; PSA: prostate specific antigen

**Figure 3:**
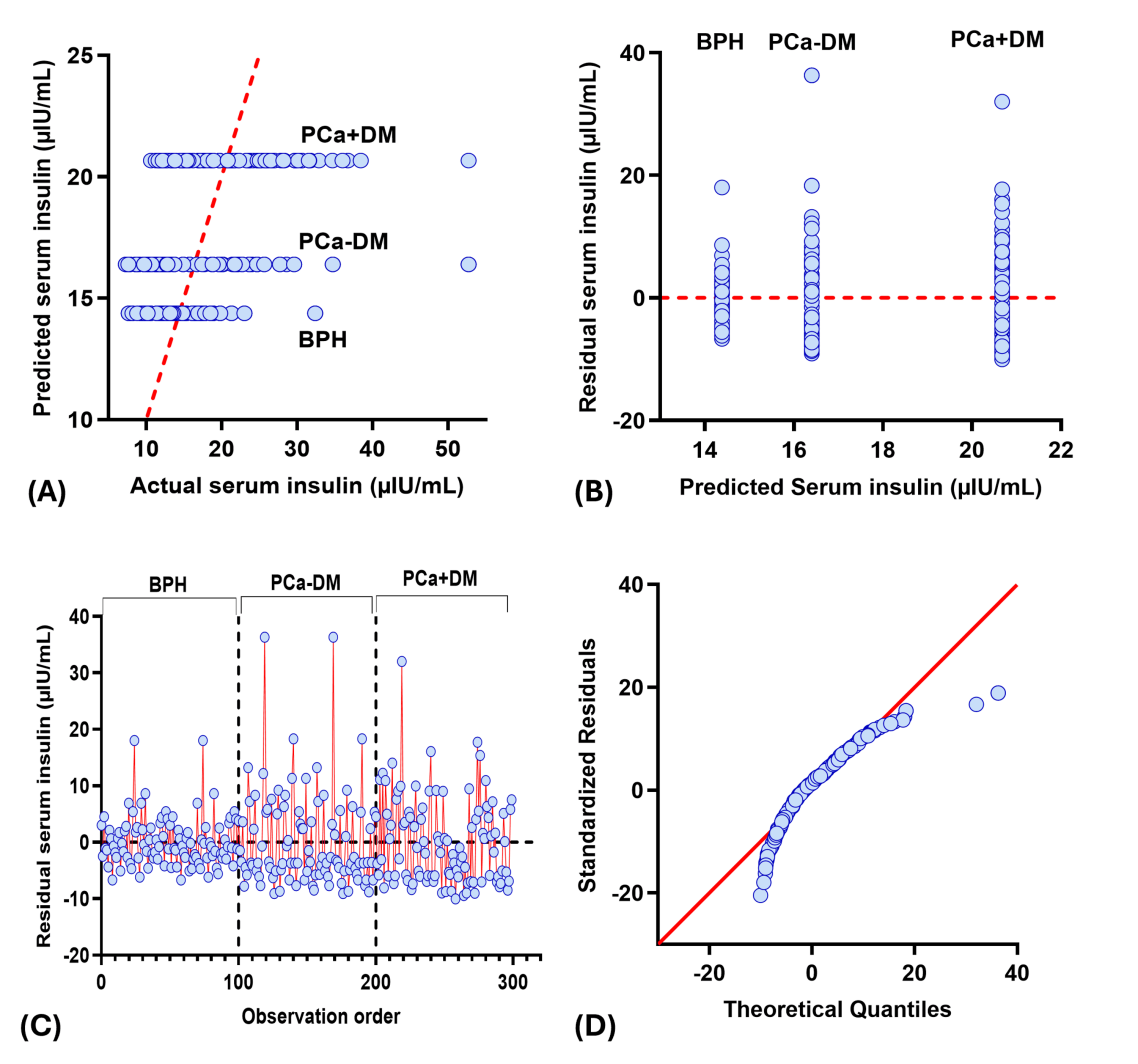
Diagnostic plot for insulin multiple regression analysis (A) Observed vs predicted plot (B) Residual vs predicted plot (C) Observation order vs residuals (D) QQ plot. PCa+DM: prostate cancer with diabetes mellitus; PCa-DM: prostate cancer without diabetes mellitus; BPH: benign prostatic hyperplasia

**Figure 4:**
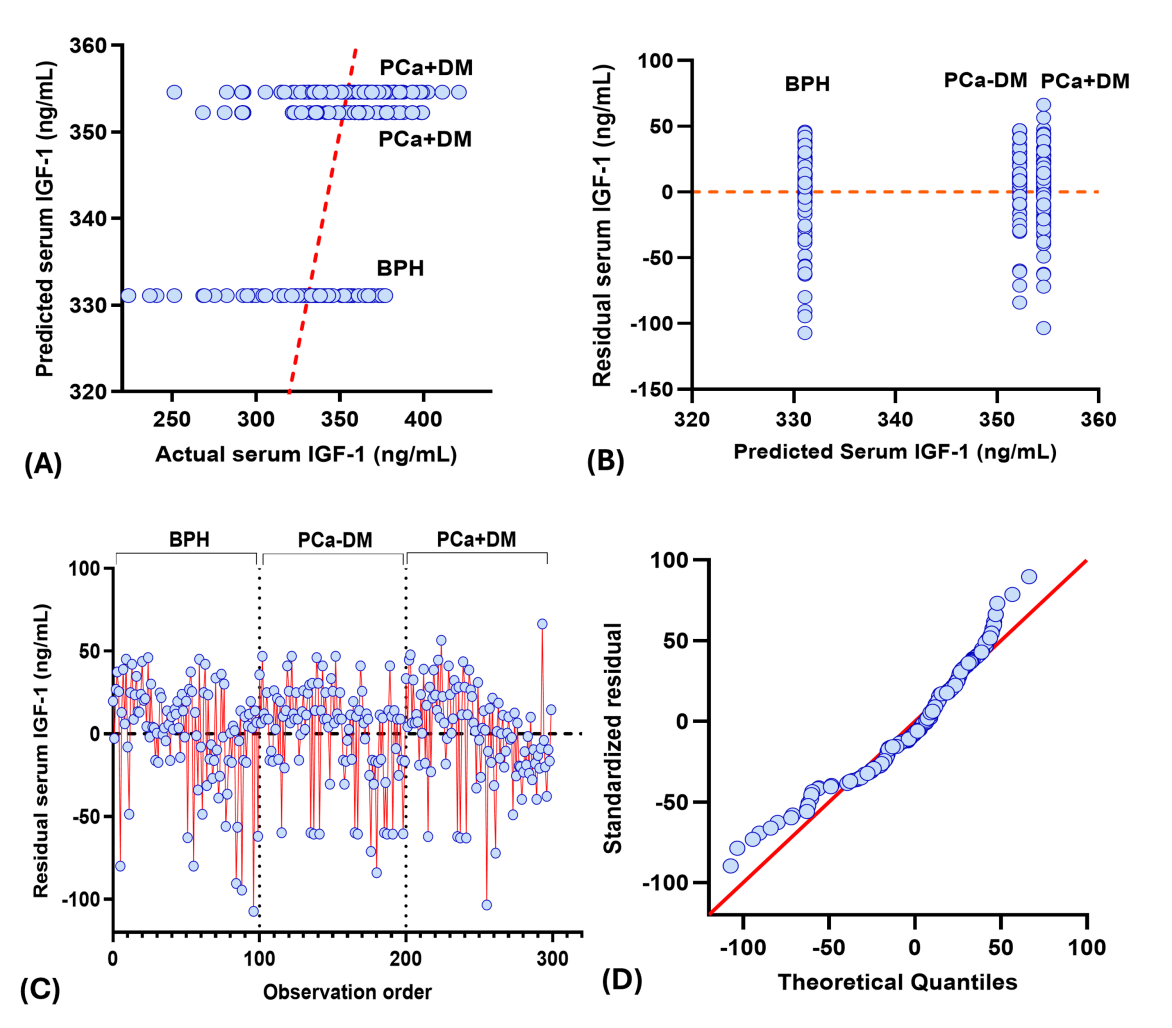
Diagnostic plot for IGF-1 multiple regression analysis (A) Observed vs predicted plot, (B) Residual vs predicted plot, (C) Observation order vs residuals, (D) QQ plot. PCa+DM: prostate cancer with diabetes mellitus; PCa-DM: prostate cancer without diabetes mellitus; BPH: benign prostatic hyperplasia; IGF-1: insulin-like growth factor-1

**Figure 5:**
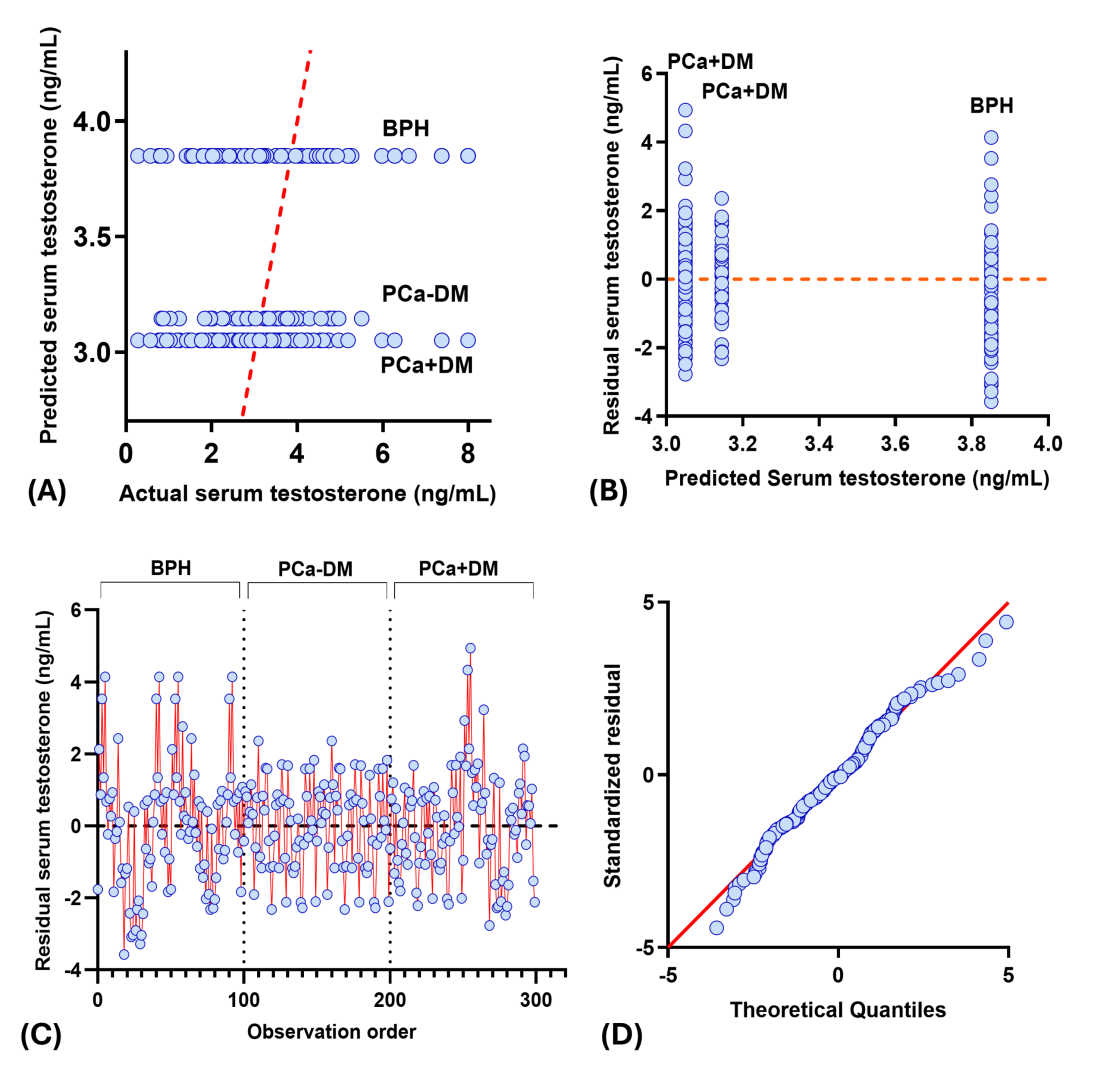
Diagnostic plot for testosterone multiple regression analysis (A) Observed vs predicted plot, (B) Residual vs predicted plot, (C) Observation order vs residuals, (D) QQ plot. PCa+DM: prostate cancer with diabetes mellitus; PCa-DM: prostate cancer without diabetes mellitus; BPH: benign prostatic hyperplasia

Actual vs predicted plot showed three different horizontal clusters, and residual vs predicted plot showed three vertical clusters corresponding to each study group, where BPH patients have the lowest predicted, actual, and residual value for PSA, insulin, and IGF-1, and vice versa for testosterone. Among the PCa group, serum insulin and IGF-1 values were higher, whereas serum PSA and testosterone values were lower in the PCa+DM group compared to the PCa-DM group. Residual vs observation order plot for PSA and insulin showed residuals clustered around the zero in the BPH group, indicating good model fit and low variability in predicted serum PSA and insulin. However, scattered residuals in both prostate cancer groups showed greater biological variability. Residuals for IGF-1 and testosterone showed a similar pattern in all study groups. QQ plots showed the normal distribution of residuals in IGF-1 and testosterone, whereas the skewed residuals were observed for PSA and insulin (Figures 2-5).

Gleason Score, cancer grade, and risk between PCa+DM and PCa-DM

**Figure 6:**
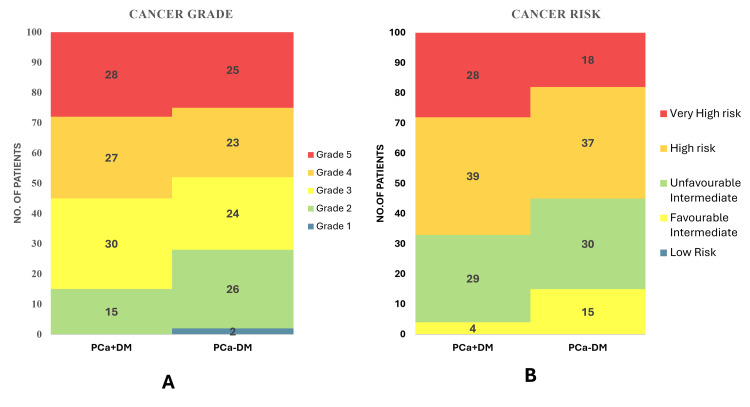
(A) Cancer Grade and (B) Cancer risk in prostate cancer patients with and without diabetes PCa+DM: prostate cancer with diabetes mellitus; PCa-DM: prostate cancer without diabetes mellitus

The comparison of GS between PCa+DM and PCa-DM showed no statistically significant difference (t=0.42; p=0.677). Similarly, the distribution of cancer grade and aggressiveness was also not significantly different (p=0.113 and p=0.322); however, the frequency of high-grade, more aggressive tumors was higher in the PCa+DM group compared to the PCa-DM. The comparison of prostate cancer risk levels between PCa+DM and PCa-DM revealed a statistically significant difference (p=0.011), with a higher frequency of high-risk tumors in the PCa+DM group. Ordinal logistic regression demonstrates that diabetes did not predict higher cancer grade; however, the negative coefficient (β) and odds ratio suggest that PCa-DM group has a reduced likelihood of high-grade cancer compared to PCa+DM group (p=0.013) (Table 4, Figure 6)

**Table 4:** Gleason score, cancer grade and risk between prostate cancer with and without diabetes Bold values represent significant results (p<0.05). ^a^Unadjusted OR; ^b^Adjusted OR for age, BMI, family history, smoking, alcohol, physical activity, diet and hypertension. PCa+DM: prostate cancer with diabetes mellitus; PCa-DM: prostate cancer without diabetes mellitus; GS: Gleason score; Ref: Reference group (PCa+DM); OR: odds ratio

	PCa+DM	PCa-DM	Unpaired t test	p-value
Gleason Score	7.72±0.82	7.77±0.87	t=0.42	0..677
Cancer Grade (No. of cases)			chi sq for trend	p-value
1	0	2	2.50	0.113
2	15	26		
3	30	24		
4	27	23		
5	28	25		
Cancer risk (No. of cases)			chi sq for trend	p-value
Low Risk	0	0	6.48	0.011
Favourable Intermediate	4	15		
Unfavourable Intermediate	29	30		
High risk	39	37		
Very High risk	28	18		
Cancer aggressiveness (No. of cases)			chi sq	p-value
More aggressive GS ≥8	55	48	0.98	0.322
Less aggressive GS≤7	45	52		
Ordinal Regression		Estimate (β)	OR	p-value
Cancer grade vs study group	Ref.	-0.380	0.68^a^	0.135
	Ref.	-0.501	0.61^b^	0.074

Relationship between metabolic parameters, hormonal parameters, and prostate cancer grade and aggressiveness in PCa+DM and PCa-DM

**Figure 7:**
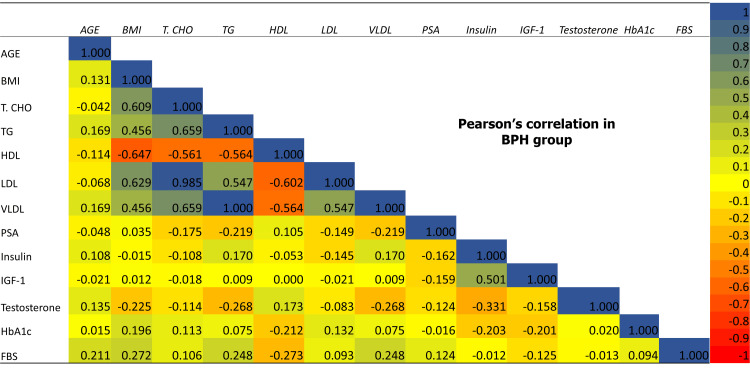
Pearson correlation of baseline, metabolic and hormonal parameters among each other in BPH group. BPH: benign prostatic hyperplasia; BMI: body mass index; HTN: hypertension, T.CHO: total cholesterol; TG: triglyceride; HDL: high density lipoprotein; LDL: low density lipoprotein; VLDL: very low density lipoprotein; HbA1c: glycated hemoglobin, FBG: fasting blood glucose; PSA: prostate specific antigen; IGF-1: insulin-like growth factor-1

**Figure 8:**
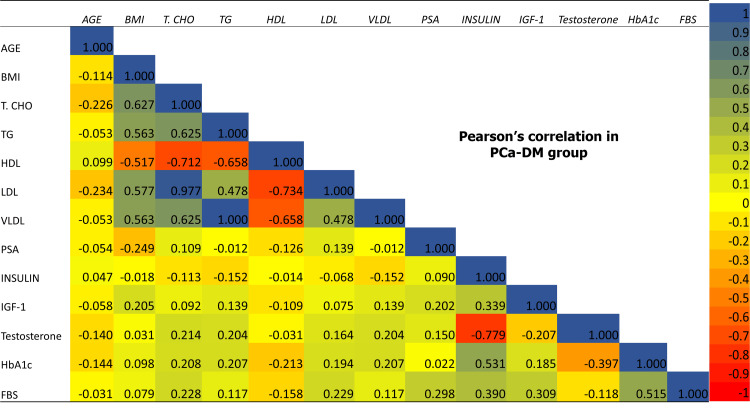
Pearson correlation of baseline, metabolic and hormonal parameters among each other in PCa-DM group. PCa-DM: prostate cancer without diabetes; BMI: body mass index; HTN: hypertension, T.CHO: total cholesterol; TG: triglyceride; HDL: high density lipoprotein; LDL: low density lipoprotein; VLDL: very low density lipoprotein; HbA1c: glycated hemoglobin, FBG: fasting blood glucose; PSA: prostate specific antigen; IGF-1: insulin-like growth factor-1

**Figure 9:**
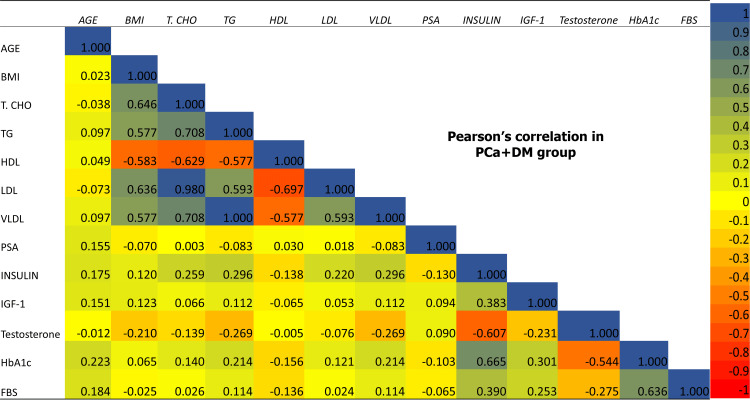
Pearson correlation of baseline, metabolic and hormonal parameters among each other in PCa+DM group. PCa+DM: prostate cancer with diabetes; BMI: body mass index; HTN: hypertension, T.CHO: total cholesterol; TG: triglyceride; HDL: high density lipoprotein; LDL: low density lipoprotein; VLDL: very low density lipoprotein; HbA1c: glycated hemoglobin, FBG: fasting blood glucose; PSA: prostate specific antigen; IGF-1: insulin-like growth factor-1

The comparison of metabolic and hormonal parameters showed a significant negative correlation of insulin and IGF-1 with testosterone in both PCa+ DM and PCa-DM groups, whereas a significant negative association of FBS and HbA1c was only found in the PCa+DM group. Lipid profile did not show any significant association with testosterone in any group. None of the study parameters showed a significant association with serum PSA levels in any of the study groups (Table 5 and Figures 7-9).

**Table 5:** Pearson’s correlation of serum PSA and testosterone with metabolic and hormonal parameters. Bold values represent significant results (p<0.05). PCa+DM: prostate cancer with diabetes mellitus; PCa-DM: prostate cancer without diabetes mellitus; BPH: benign prostatic hyperplasia; BMI: body mass index; HTN: hypertension, T.CHO: total cholesterol; TG: triglyceride; HDL: high density lipoprotein; LDL: low density lipoprotein; VLDL: very low density lipoprotein; HbA1c: glycated hemoglobin, FBG: fasting blood glucose; PSA: prostate-specific antigen; IGF-1: insulin-like growth factor-1

Variable		PSA			Testosterone		
		PCa+DM	PCa-DM	BPH	PCa+DM	PCa-DM	BPH
AGE	r-value	0.155	-0.054	-0.048	-0.011	-0.140	0.135
	p-value	0.124	0.594	0.635	0.914	0.165	0.181
BMI	r-value	-0.070	-0.249	0.035	-0.210	0.031	-0.225
	p-value	0.488	0.012	0.729	0.036	0.759	0.024
T. CHO	r-value	0.002	0.109	-0.175	-0.138	0.214	-0.114
	p-value	0.984	0.280	0.081	0.171	0.031	0.259
TG	r-value	-0.083	-0.012	-0.219	-0.268	0.204	-0.268
	p-value	0.412	0.906	0.029	0.007	0.042	0.007
HDL	r-value	0.029	-0.126	0.105	-0.005	-0.031	0.173
	p-value	0.771	0.212	0.230	0.961	0.760	0.085
LDL	r-value	0.018	0.139	-0.149	-0.075	0.164	-0.083
	p-value	0.859	0.168	0.139	0.459	0.103	0.412
VLDL	r-value	-0.083	-0.012	-0.219	-0.268	0.204	-0.268
	p-value	0.412	0.906	0.029	0.007	0.042	0.007
FBG	r-value	-0.065	0.298	0.124	-0.274	-0.117	-0.013
	p-value	0.521	0.003	0.219	0.006	0.246	0.263
HbA1c	r-value	-0.103	0.022	-0.016	-0.543	-0.39	0.020
	p-value	0.308	0.828	0.874	<0.00001	0.700	0.843
PSA	r-value	1	1	1	0.090	0.149	-0.124
	p-value	<0.00001	<0.00001	<0.00001	0.373	0.139	0.210
Insulin	r-value	-0.129	0.090	0.162	-0.607	-0.779	-0.331
	p-value	0.201	0.373	0.107	<0.00001	<0.00001	0.0007
IGF-1	r-value	0.093	0.201	-0.159	-0.230	-0.206	-0.158
	p-value	0.357	0.045	0.114	0.021	0.040	0.116
Testosterone	r-value	0.090	0.149	-0.124	1	1	1
	p-value	0.373	0.139	0.210	<0.00001	<0.00001	<0.00001

**Figure 10:**
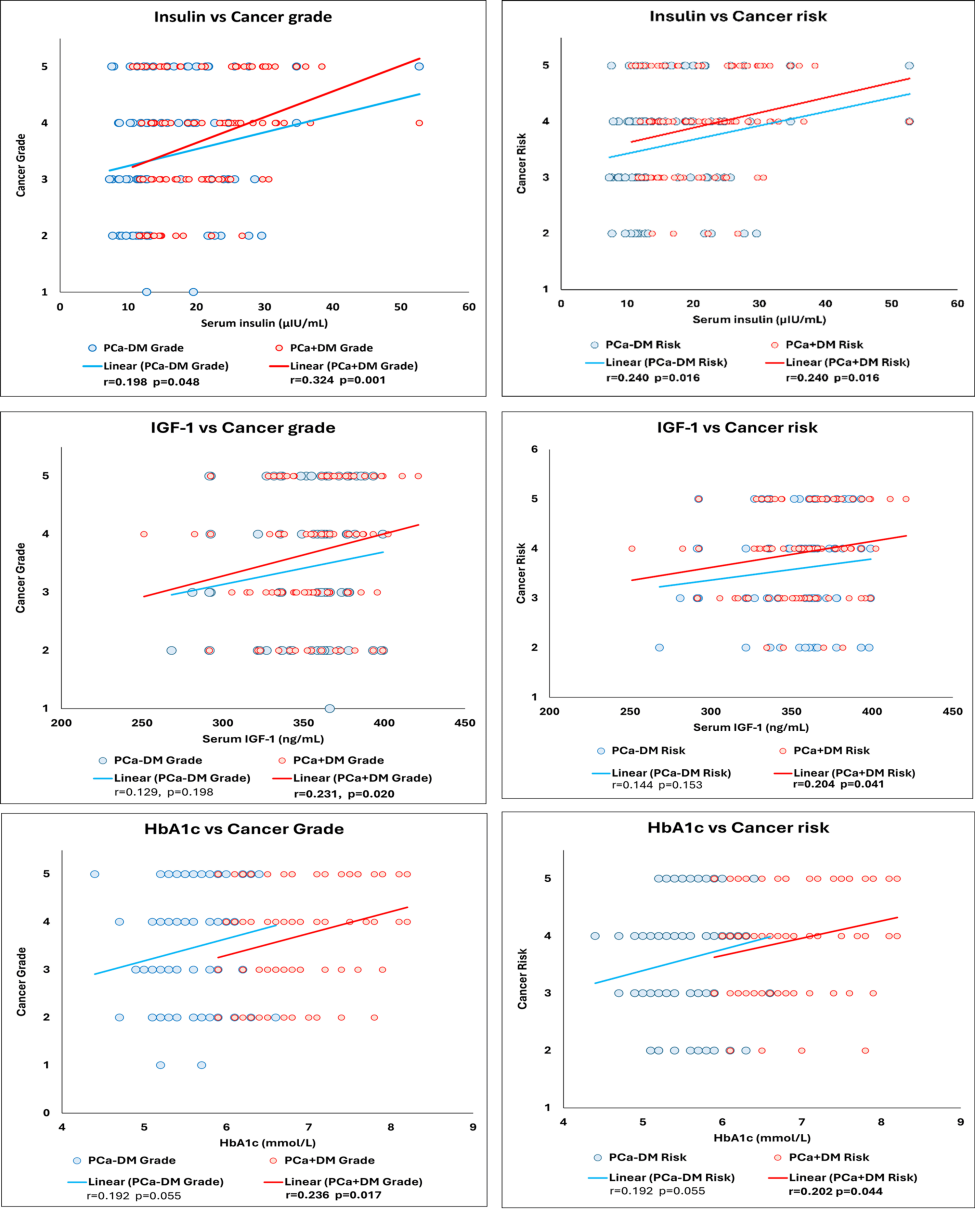
Scatter plot showing correlation of serum insulin, IGF-1 and HbA1c with cancer grade and risk in prostate cancer with and without diabetes. P value is calculated using spearman's correlation. p value <0.05 is considered statistically significant. PCa-DM: prostate cancer without diabetes; PCa+DM: prostate cancer with diabetes, IGF-1:insulin-like growth factor 1; HbA1c: glycated hemoglobin

The comparison of metabolic and hormonal parameters showed a significant positive association of total cholesterol, TG, VLDL, and LDL, and a significant negative association of HDL with cancer grade and risk in the PCa-DM group, but not in the PCa+DM. Insulin was significantly positively associated with cancer grade and risk in both PCa+DM and PCa-DM groups. IGF-1 and HbA1c showed a significant positive association with cancer grade and risk in the PCa+DM group only (Table 6, Figure 10)

**Table 6:** Spearman Correlation of metabolic and hormonal markers with prostate cancer grade and risk in diabetic and non-diabetic prostate cancer Bold values represent significant results (p<0.05). PCa+DM: prostate cancer with diabetes; PCa-DM: prostate cancer without diabetes; BMI: body mass index; HTN: hypertension, T.CHO: total cholesterol; TG: triglyceride; HDL: high density lipoprotein; LDL: low density lipoprotein; VLDL: very low density lipoprotein; HbA1c: glycated hemoglobin, FBG: fasting blood glucose; PSA: prostate specific antigen; IGF-1: insulin-like growth factor-1

	Cancer Grade				Cancer Risk			
	PCa+DM		PCa-DM		PCa+DM		PCa-DM	
	r-value	p-value	r-value	p-value	r-value	p-value	r-value	p-value
AGE	0.089	0.376	-0.176	0.080	0.084	0.405	-0.115	0.25
BMI	0.025	0.803	0.168	0.093	0.012	0.909	0.181	0.072
T. CHO	0.054	0.591	0.359	0.0002	0.003	0.970	0.389	<0.0001
TG	0.083	0.408	0.207	0.039	0.037	0.710	0.199	0.047
HDL	-0.071	0.478	-0.225	0.024	-0.022	0.800	-0.225	0.024
LDL	0.033	0.747	0.341	0.0005	0.023	0.813	0.376	0.0001
VLDL	0.083	0.408	0.207	0.038	0.037	0.711	0.199	0.047
PSA	0.092	0.361	0.271	0.006	0.090	0.370	0.343	0.0004
Insulin	0.324	0.001	0.198	0.048	0.240	0.016	0.240	0.016
IGF-1	0.231	0.020	0.129	0.198	0.204	0.041	0.144	0.153
Testosterone	-0.171	0.087	-0.039	0.702	0.131	0.191	0.091	0.363
FBS	-0.030	0.762	0.287	0.003	-0.09	0.32	0.274	0.005
HbA1c	0.236	0.017	0.192	0.055	0.202	0.044	0.192	0.55

## Discussion

This study investigated the influence of diabetes on metabolic and hormonal profiles in prostate cancer, as patients with prostate cancer and diabetes had a 29% higher prostate cancer-specific mortality and a 37% higher all-cause mortality compared to patients with prostate cancer but without diabetes [15]; however, many studies paradoxically suggest a protective effect of diabetes on prostate cancer incidence.

Age is considered to be a risk factor for both BPH and prostate cancer, and with increasing age, the risk of development of prostate cancer gradually increases. The linkage of age with prostate cancer screening, diagnosis, and treatment had already been established in some community-based studies, which might be due to damaged prostate blood supply and alteration in levels of sex hormones with age [16]. Our results are in line with these studies, where we observed a higher mean age in the prostate cancer groups compared to BPH. This difference might be due to the fact that the development of prostate cancer occurs at an older age compared to BPH. However, we did not find any difference in the mean age of patients and association with cancer aggressiveness in prostate cancer groups with and without diabetes. In contrast to our findings, a study reported an increased risk of more aggressive prostate cancer in older men [17]. Increased BMI has been associated with numerous cancers, including prostate cancer, with increased adiposity leading to increased mortality risk of prostate cancer. According to the meta-analysis by Cao and Ma, an increase of 5 kg/m^2^ in BMI led to a 20% higher risk of prostate cancer mortality [18]. However, in the present study, we did not observe any significant difference between the value of BMI among the BPH, PCa+DM, and PCa-DM groups.

Dyslipidemia is directly associated with mortality in elderly prostate cancer patients. In the present study, the mean value of total cholesterol, TG, LDL, and VLDL was found to be higher in patients with prostate cancer and diabetes as compared to those with only prostate cancer or with BPH. However, the mean difference did not reach statistical significance. HDL levels were significantly decreased in both prostate cancer groups compared to BPH controls. Similar findings were reported in some previous studies [19]. Our study also reported an association of a deranged lipid profile with higher cancer grade and risk. However, the association is found to be significant only in the non-diabetic group.

Some studies also reported serum lipids to be a modifiable factor that influences the risk of prostate cancer recurrence. However, they found no evidence to support their findings. Possible association between deranged lipid profile and prostate cancer recurrence might be due to multiple biological pathways through which serum lipids might affect prostate cancer progression [20,21]. Another systemic analysis studying the influence on lipid profile reported significantly higher levels of total cholesterol and TG after one year of androgen deprivation therapy (ADT), suggesting a vital role of cholesterol in promoting prostate cancer [22].

Hyperglycemia may promote tumor growth and resistance to cancer therapy. Elevated HbA1C may worsen cancer outcomes even in non-diabetic prostate cancer patients, as it has been associated with all-cause mortality in both diabetic and non-diabetic populations [23]. We have reported similar findings of elevated FBS and HbA1c in both prostate cancer compared to BPH, with significant differences, particularly in the PCa+DM group. We also reported a significant association of FBS and HbA1c with higher-grade and high-risk prostate cancer in the PCa+DM group, which is consistent with the findings of Lutz et al. [9].

Diabetes and prostate cancer share a complex bidirectional relationship where hyperglycemia due to diabetes causes hypogonadism and low serum testosterone levels, which can lead to diabetes [24]. This inverse association is suggested to be due to long-term diabetes, in which increased levels of sex hormone-binding globulin (SHBG) could reduce the testosterone levels, or due to elevated levels of growth factors among patients with diabetes [25]. We observed reduced testosterone levels in both prostate cancer groups compared to the BPH control, and a non-significant negative association of testosterone with prostate cancer Gleason score and grade. Some recent studies also linked lower testosterone levels with increased severity of prostate cancer [18,26]. In contrast to our findings, one study cohort reported an increased risk of prostate cancer with elevated serum testosterone levels [27].

Our study reported significantly elevated serum PSA levels in both prostate cancer groups compared to BPH. However, on comparing both prostate cancer groups, we observed a decrease in the serum PSA levels in the PCa+DM group than in the PCa-DM group. The paradoxically lower PSA levels in the PCa+DM group (22.38 vs 39.72 ng/mL) may reflect the suppressive effect of low testosterone on PSA expression rather than less aggressive disease. This diagnostic challenge could lead to delayed detection and contribute to the observed higher-grade tumors in patients with diabetes, representing a detection bias rather than biological aggressiveness. Similar findings were reported by the National Health and Nutritional Examination Survey (NHANES) II, in which a 21.6% decrease in mean PSA levels was observed in men with diabetes compared to men without diabetes, and the difference increases with the increase in years since diagnosis of diabetes. These findings might also explain the lower incidence of prostate cancer in men with diabetes, as fewer men with diabetes might proceed to prostate biopsy due to diagnostic bias [28].

Hyperinsulinemia is an important characteristic of diabetes, which enhances prostatic epithelial cell proliferation, thus increasing tumor migration and invasiveness in diabetics. Insulin binding to its receptor initiates cascades of reactions, including activation of tyrosine Kinase receptor, phosphoinositide 3-kinase (PI3K)-Akt, and mitogen-activated protein kinase (MAPK) signaling pathway. The tyrosine kinase receptor also responds to IGF-1 after its binding with the IGF-1 receptor. Hence, IGF-1 receptor expression in the tumor cells might cause progression to a lethal phenotype, being more sensitive to IGF signaling [29]. In our study, elevated levels of insulin and IGF-1 in the PCa-DM group are positively correlated with the prostate cancer grade and risk, supporting the hypothesis that the mitogenic and oncogenic roles of insulin and IGF-1 might be associated with cancer aggressiveness in the diabetic group. Similar findings were also reported by previous studies [30]. In contrast, some studies suggested that long-term diabetes transitions towards reduced levels of Insulin, IGF-1, and testosterone over time, partially explaining the lower incidence of prostate cancer in men with diabetes. Also, the reduced risk of prostate cancer in men with diabetes could also be due to the involvement of medications for diabetes, such as metformin [31,32]. Results of our multiple linear regression analysis also indicate that the presence of both prostate cancer and diabetes mellitus may amplify biochemical alterations more than prostate cancer alone.

We did not find significant difference between mean Gleason scores of both prostate cancer groups, which appears to suggest the grade distribution of prostate cancer was broadly similar between the two groups, regardless of diabetic status; however we observed greater frequency of higher Gleason score, cancer grade, and more aggressive prostate cancer in the diabetic group indicating that the presence of diabetes mellitus might influence the Gleason grading of prostate cancer in the study population. Similar findings of more aggressive prostate cancer and higher Gleason score, and increased lymph node metastasis incidences were reported by Kim et al. [33] and Lutz et al. [9]. In contrast, a recent study examining the relationship between prostate cancer and type 2 diabetes mellitus at two different time periods following different screening guidelines reported the incidence of smaller, less aggressive prostate cancer in men with diabetes [10]. The protective effect of diabetes on early prostate cancer development was also reported by a recent meta-analysis, but they did not report any association with advanced prostate cancer stages. This discrepancy in the reported data might be due to different prostate cancer detection and risk estimation methods [34]. Moreover, as shown in our study, patients with prostate cancer and diabetes had lower PSA levels; hence, reduced sensitivity of PSA might be the reason behind the reported protective effect or lower incidence, indicating a diagnostic bias rather than biological protection.

Limitations of the study

Our study may have certain limitations. A major limitation is the absence of data on diabetes duration, glycemic control (only a single HbA1c measurement), and antidiabetic medications, particularly metformin and other confounders such as drug dosing, socioeconomic status, etc. This represents a critical confounding factor as: (i) early diabetes is characterized by hyperinsulinemia while advanced diabetes shows insulin deficiency, (ii) metformin has established anti-cancer properties, and (iii) diabetes duration affects testosterone and PSA levels. Without adjusting for these factors, we cannot attribute observed differences to diabetes per se versus specific diabetes-related characteristics or treatments.

Moreover, bias from daily dietary habits, smoking status, alcohol consumption, and physical activity may exist and could possibly affect the risk of prostate cancer. Our study is single-centric, particularly including participants from North India (Uttar Pradesh region), which limits the applicability of the findings to a limited geographic region. Also, the study design cannot determine the temporality or direction of influence. Additionally, the small sample size with no systematic follow-up of patients may limit statistical power. We can furthermore not exclude that the difference in age at diagnosis of prostate cancer in patients with and without diabetes might have influenced our results.

Future studies including a larger sample size and varied ethnicity, along with information on diabetes duration and medication usage, may be useful in validating the reported results.

## Conclusions

Our results emphasize the strong influence of diabetic status on serum PSA as well as glycemic, lipid, and hormonal parameters, particularly HDL, HbA1c, FBS, insulin, IGF-1, and testosterone. Hyperglycemia, hyperinsulinemia, and dyslipidemia are the characteristics of diabetes, all of which are associated with an elevated risk of the development of higher-grade, more aggressive prostate cancer. Patients with prostate cancer and diabetes have lower PSA and testosterone levels and higher serum FBS, HbA1c, insulin, and IGF-1 levels, along with a deranged lipid profile, leading to observed greater cancer grade and high risk in men with diabetes.

Our study suggests that men with diabetes who are diagnosed with prostate cancer present with higher tumor grades at diagnosis. However, whether this reflects true biological aggressiveness enhanced by diabetes or delayed detection due to masking of prostate cancer diagnosis due to lower PSA levels cannot be determined from our data. Longitudinal studies with adjustment for detection bias and diabetes characteristics are needed to establish whether diabetes truly has a deteriorative effect on prostate cancer outcome and worsens prostate cancer prognosis.
